# Quantification of Viable *Salmonella* by Propidium Monoazide Real-Time PCR After Long-Term Storage of Peanut Products

**DOI:** 10.3390/microorganisms12122640

**Published:** 2024-12-19

**Authors:** Aline M. von Hertwig, André A. Pereira, Dionisio Pedro Amorim Neto, Maristela S. Nascimento

**Affiliations:** Faculty of Food Engineering, University of Campinas, Campinas 13083-862, Brazil; alinemorgan.morgan@gmail.com (A.M.v.H.); andreakino1710@gmail.com (A.A.P.); amorimnetodp@gmail.com (D.P.A.N.)

**Keywords:** propidium monoazide (PMA), viable but non-culturable cells (VBNC), *Salmonella*, low-moisture food, peanuts

## Abstract

In this study, the performance of quantitative PCR, combined or not with propidium monoazide (PMA), to recover *Salmonella* from peanut products after different storage times was evaluated. The samples were inoculated with 5–6 log cfu g^−1^ of *Salmonella* Typhimurium ATCC 14028 and stored at 28 °C for up to 540 d. The correlation between the threshold cycle number (Ct) and the colony-forming units (cfu) was obtained by a standard curve, which showed a linear correlation (R^2^ = 0.97). The highest counts were recovered by qPCR (*p* < 0.05); however, it quantified both viable and non-viable cells. For roasted peanuts, a significant difference (*p* < 0.05) between qPCR-PMA and the culture method was verified only for samples stored for 30 d, i.e., 2.8 versus 4.0 log cfu g^−1^. Further, there was no VBNC status in the roasted peanuts, even after long-term exposure to desiccation stress. For peanut-based products, after 540 d, only *paçoca* showed a significant difference (*p* < 0.05) among the three methods evaluated. In peanut brittle, qPCR-PMA detected 1.5 log cfu g^−1^, while, in the culture method, *Salmonella* was recovered in 1 g. The pathogen was below the detection limit in *pé-de-moça* either by plate count or qPCR-PMA. Therefore, qPCR-PMA shows potential for use in quantifying *Salmonella* in peanut products.

## 1. Introduction

*Salmonella* Typhimurium stands out as one of the most prevalent *Salmonella* serotypes [1]. It is responsible for several global outbreaks each year that involve a variety of food categories, including low-moisture foods (LMFs, water activity (a_w_) ≤ 0.85) [2]. In 2022, *S.* Typhimurium was responsible for an outbreak associated with chocolate, which resulted in 455 cases globally. This serotype was also the etiologic agent of a multi-country outbreak linked to Brazil nuts in 2019–2020 in Europe, with 123 cases and one death [3]. In 2018, in the USA, *S.* Typhimurium caused 14 cases of illness after the consumption of dried coconut [4]. In addition, it was responsible for the largest outbreak linked to peanut butter in 2008–2009 in the USA, with 714 cases and nine deaths [5]. There are no data available in Brazil on salmonellosis outbreaks involving LMF. However, *S.* Typhimurium has a high incidence in meat and poultry products in this country [6,7].

A possible reason for the occurrence of outbreaks in LMF is the ability of *Salmonella* to survive for long periods in this food category. In a previous study, the pathogen was recovered from roasted peanuts after 420 d at 28 °C [8,9]. For long-term survival, *Salmonella* cells undergo several adaptations. During desiccant stress, the cells induce adaptive physiological responses in order to maintain turgor pressure, designed to increase the concentrations of solutes and osmoprotectors [10]. Another response to a low a_w_ environment has been reported in *Salmonella* Enteritidis, this being the degradation of ribosomal RNA molecules [11]. Finally, under desiccation, a part of the *Salmonella* population may become viable but non-culturable cells (VBNC) [10,12]. In this state, bacterial cells enter a physiologically dormant condition, with only 5% of their genome being transcribed [11]. Despite this, the cells retain their virulence potential, becoming a food safety issue [13]. This phenomenon can result in the underestimation of *Salmonella* in LMF and, consequently, represent a risk to the consumer’s health, since VBNC cannot be detected by the traditional cultural method [14]. Furthermore, the ability to survive under desiccant stress contributes to cross-protection, resulting in infectious doses as low as 0.04 cfu g^−1^ [15]. The high heat resistance of *Salmonella* in LMF has also been reported [10,16].

In the last few decades, molecular methods such as real-time PCR (qPCR) have been widely used for quantitative detection; they are considered a faster, more sensitive and less laborious technique than traditional ones. However, the great limitation of this technique in quantifying pathogens in food is its inability to differentiate DNA from viable and non-viable cells. Therefore, the combination of two techniques can potentially be used to detect only viable bacteria. The former is based on the detection of mRNA using reverse transcription (RT) qPCR [17,18,19]. However, this detection technique requires the expression of target genes, which may vary under stress conditions. Furthermore, RNA is very sensitive to degradation in complex matrices, such as food. The latter technique is based on the detection of DNA from cells with intact membranes. For this, before qPCR amplification, a viability discrimination step is performed. For this, two known molecules can be used, ethidium monoazide (EMA) and propidium monoazide (PMA), derived from ethidium bromide and propidium iodide, respectively. The major concern associated with EMA is that this molecule can penetrate and is toxic to viable bacteria [20,21]. The action of PMA is based on the integrity of bacterial cells, as this dye only penetrates dead cells or those with a compromised membrane [21,22]. PMA binds covalently to DNA, and the presence of the monoazide group allows the dye to cross-link with DNA after exposure to strong visible light. Light leads to the formation of a highly reactive nitrene radical, which inhibits the PCR amplification of dead cell DNA [21,23].

Thus far, the qPCR-PMA method has been successfully implemented to detect a wide variety of microorganisms in food matrices [24,25]. However, there are few studies that have evaluated the detection of *Salmonella* in LMF through qPCR-PMA [26,27,28] and none using peanuts as an LMF matrix. In addition, as mentioned before, environmental stresses such as desiccation can induce bacteria to enter a viable but non-culturable state. However, traditional plate enumeration techniques are not able to detect such injured cells; therefore, a culture-independent enumeration methodology is essential to assess the effects of control strategies and provide reliable data for risk assessment. Thus, the feasibility of quantitative PCR (qPCR) combined with PMA to determine *Salmonella* Typhimurium ATCC 14028 in roasted peanuts after short- and long-term storage, and in three peanut-based products after long-term storage, was evaluated.

## 2. Materials and Methods

### 2.1. Inoculum

The target strain for this study was *Salmonella* Typhimurium ATCC 14028. The strain was stored in a biofreezer (−80 °C) in trypticase soy broth (TSB, Difco, Sparks, MD, USA) with 15% (*v*/*v*) glycerol. The culture was placed in TSB at 37 ± 1 °C for 18 ± 2 h. After this period, the culture was streaked on trypticase soy agar (TSA, Difco) and incubated at 37 ± 1 °C for 18 ± 2 h, with subsequent storage at 4 ± 1 °C. To prepare the inoculum, the culture was stored at 4 ± 1 °C and was cultivated twice in TSB at 37 ± 1 °C for 18 ± 2 h. Then, the broth was centrifuged at 7740× *g* for 7 min at 4 °C (J2-21, Beckman, Chaska, MN, USA). The supernatant was then discarded and the biomass obtained was resuspended in saline solution (0.85% *w*/*v*), to achieve a final concentration of 10^8^ cfu mL^−1^. The number of cells in the suspension was determined by serial dilutions, followed by pour plating on TSA, with incubation at 37 ± 1 °C for 24 ± 2 h.

### 2.2. Inoculation and Storage

Roasted peanut kernels (a_w_ = 0.43) and three peanut-based products (peanut brittle (a_w_ = 0.30), *paçoca* (a_w_ = 0.40) and *pé-de-moça* (a_w_ = 0.68)) were used as LMF matrices. The samples were collected from retail stores in Campinas, Brazil and previously tested for *Salmonella* [29]. Only negative samples were used in this study.

The peanut-based products were previously crushed in a blender (Blendetec, Orem, UT, USA) under 30 s cycles to obtain a homogeneous sample. Then, the food matrices (1200 ± 1 g) were inoculated through spraying with 0.38% *v*/*w* of a *Salmonella* suspension (10^8^ cfu mL^−1^), supplemented with 0.6% Tween 80 (Merck, Darmstadt, Germany) and homogenized by hand for 2 min [24]. After this, the samples were transferred to aluminum screen trays and remained in a biosafety cabinet (Vecco, Campinas, Brazil) for up to 20 min to bring the a_w_ back to the original level [9]. The samples were divided into three 400 g portions and transferred to sterile plastic bags (Twirl’em, Labplas, Montreal, QC, Canada). The *Salmonella* concentration immediately after sample inoculation was around 5–6 log cfu g^−1^.

To avoid significant fluctuations in the a_w_ values throughout the storage time, desiccators containing potassium carbonate solution (a_w_ 0.43) for *paçoca* and roasted peanuts and magnesium chloride (a_w_ = 0.32) for peanut brittle were used [9], while *pé-de-moça* was kept in a desiccator without any isotherm solution. The desiccators were stored in an incubator (FANEM, São Paulo, Brazil) at 28 ± 1 °C for up to 540 d. The experiment was repeated twice.

The storage temperature chosen for this study was based on meteorological data from INMET, which indicated that this is the average temperature for most of Brazil [30].

The a_w_ values of the samples were determined in duplicate at 25 ± 1 °C with a water activity meter–hygrometer (Aqualab PRE CAP, Decagon Device, Pullman, WA, USA).

### 2.3. Salmonella Enumeration

The *Salmonella* population was determined after 0, 30, 60, 120, 180 and 540 d for roasted peanuts and after 540 d for the peanut-based products by plate counting. Simultaneously, DNA extraction was performed for subsequent quantification in qPCR. Within each trial, the PCR analyses were run in triplicate and the plate count once.

#### 2.3.1. Culture Method

Ten grams (10 g) of each sample were transferred into Schott bottles containing 90 mL of 0.1% (*w*/*v*) peptone water (Acumedia, Saint Johns, MI, USA) and homogenized on a shaker (Lab-Line Orbit Environ-3527 Shaker, LabLine Instruments, Melrose Park, IL, USA) for 1 min. Serial dilutions in 0.1% peptone water (Acumedia), followed by pour plating on xylose lysine deoxycholate (XLD) agar (Acumedia) and incubation at 37 ± 1 °C for 24 ± 2 h, were performed. Five typical colonies were confirmed by biochemical and serological tests [29]. The *Salmonella* population was calculated based on the percentage of colonies tested that were confirmed as *Salmonella*. The results were expressed as colony-forming units. For samples where the counts were below the limit of quantification (1 log cfu g^−1^), a qualitative analysis was performed. For this, 1 g of sample was added to 9 mL of buffered peptone water (BPW, Acumedia) and incubated for 18–20 h at 37 ± 1 °C. Then, a loopful (10 µL) of the culture medium was streaked on XLD agar and incubated at 37 ± 1 °C for 24 ± 2 h [29]. The results were expressed as presence or absence in 1 g.

#### 2.3.2. PMA Treatment

One milliliter (1 mL) of the first decimal dilution was prepared as described in Section 2.3.1 and transferred to a microtube and mixed with 10 µL of monoazide propidium (PMA, Biotium, Fremont, CA, USA) to obtain a final concentration of 25 µM. Then, the microtubes were kept in a dark box for 5 min [31]. After this, they were placed in a container with crushed ice and exposed to a 500 W halogen lamp, at a distance of 20 cm for 10 min, for the photoactivation of the compound (adapted from Duarte et al. [14]). The microtubes were rotated every 60 s for homogeneous exposure to light, ensuring the complete binding of the intercalant with non-viable cells’ DNA [31]. Another 1 mL not treated with PMA was used to compare the results obtained by qPCR.

#### 2.3.3. DNA Extraction

For DNA extraction, the PureLink^®^ Genomic DNA Mini Kit (Applied Biosystems, Carlsbad, CA, USA) was used. Aliquots of 1 mL of samples treated and untreated with PMA were centrifuged at a maximum speed of 22,000× *g* for 1 min (J2-21, Beckman), and the supernatant was discarded. Then, the pellet was resuspended in 180 µL of the Genomic Digestion Buffer solution and homogenized with 20 µL of Proteinase K (Merck, Germany). The suspension was bathed at 55 °C for 2 h; then, 20 µL of RNase was added and it was left at room temperature. After 2 min, 200 µL of lysis/binding buffer and 200 µL of absolute ethanol were added. The resulting solution was transferred to the kit column and centrifuged at 10,000× *g* for 1 min. The column was transferred to a new kit tube, and then 500 µL of the wash buffer solution was added. The column was again centrifuged at 10,000× *g* for 1 min and transferred to a new tube. Finally, the DNA was resuspended in 60 µL of elution buffer. The concentrations and quality of the DNA in the samples were determined in a Nanodrop 2000 (Fisher Scientific, Vienna, Austria). The samples were stored at −20 °C for subsequent use.

#### 2.3.4. Salmonella qPCR Enumeration

The qPCR reactions were performed in a real-time thermocycler (7500 FAST Real-Time PCR System, Applied Biosystems). The TaqMan Universal PCR Master Mix kit (Applied Biosystems) was used to amplify the *Salmonella* DNA. For the detection of 284 base pairs (bp)/(amplicon) of the *invA* gene of *S. enterica*, primer pairs 139–141 (139: 5′-GTGAAATTATCGCCACGTT CGGGCAA-3′ and 141: 5′-TCATCGCACCGTCAAAGGAACC-3′) [32,33] and the TaqMan probe 5′-FAM-5CCAGGCTTCCAGTACGCTTCGCCGTTCGCCTGG-3′ [33] were used. To the reactions, 0.4 µL of each primer and 0.2 µL of the probe were used, and nuclease-free Milli-Q water was added to complete the final volume to 20 µL. Then, 2 µL of extracted DNA, treated or untreated with PMA, was added. The thermocycling parameters used were 95 °C/12 min for polymerase activation, followed by 45 cycles of denaturation at 95 °C/20 s; 54 °C/30 s of annealing; and extension at 72 °C/30 s [33]. Negative controls consisting of reference strains and ultrapure distilled water free of DNase/RNase were used in each qPCR run. An internal amplification control was applied as an indicator of false-negative results due to the interference of inhibitors in the food matrices.

All tests were performed in triplicate. At the end of the process, the fluorescence emitted was expressed as a threshold cycle number (Ct) and converted by linear regression into cfu g^−1^, based on the data obtained in the standard curve test, as described below.

### 2.4. Standard Curve for Quantification

To build the standard curve, two independent trials with 10 runs were conducted. Serial 10-fold dilutions of *Salmonella* cultivated in TSB (Difco) were prepared to obtain counts between 10^1^ and 10^8^ cfu mL^−1^. Then, DNA was extracted from each sample dilution as described in Section 2.3.3. Concurrently, the bacterial load was enumerated by plate pouring on TSA (Difco). The curve was built by plotting the plate count (log cfu mL^−1^) against the Ct (adapted from Josefsen et al. [31]). The slope of the curve was used to determine the amplification efficiency (E) using Equation (1) [34]:

E = [10^(−1/slope)^ − 1] × 100 (1)

### 2.5. Statistical Analysis

An analysis of variance (ANOVA) and Tukey’s test were performed to verify the significant differences (*p* < 0.05) among the *Salmonella* enumeration methods, using the software STATISTICA 7.0 (Statsoft, Tulsa, OK, USA). The standard curve was obtained by linear regression between the observed Ct and the predicted log correlation using Microsoft Excel Version 2411 (Microsoft, Los Lunas, NM, USA). The Bland–Altman analysis of the agreement limits between qPCR-PMA and the culture method was performed with GraphPad Prism version 8.

## 3. Results

**Figure 1:**
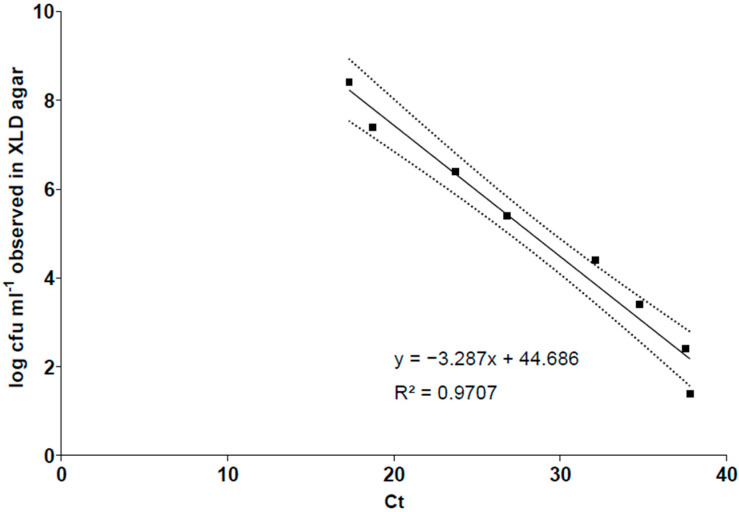
Linear relationship between the threshold cycle (Ct) and the bacterial count (log cfu mL^−1^). y = Ct observed and x = log cfu mL^−1^. The solid line represents the linear fit and the dashed lines represent the 95% confidence interval bands.

Initially, a standard curve (Figure 1) was built from the Ct and log cfu mL^−1^ of the viable cells from a pure culture of *S.* Typhimurium ATCC 14028. The efficiency (E) was 101% with a slope of −3.287, and the square regression coefficient (R^2^) was 0.971, i.e., there was a good linear correlation between the Ct and log cfu. The level of *Salmonella* was expressed by the equation of the straight line shown in Figure 1. The lowest quantification level obtained in the standard curve was 1.4 log cfu mL^−1^, corresponding to a Ct of 37.8.

**Figure 2:**
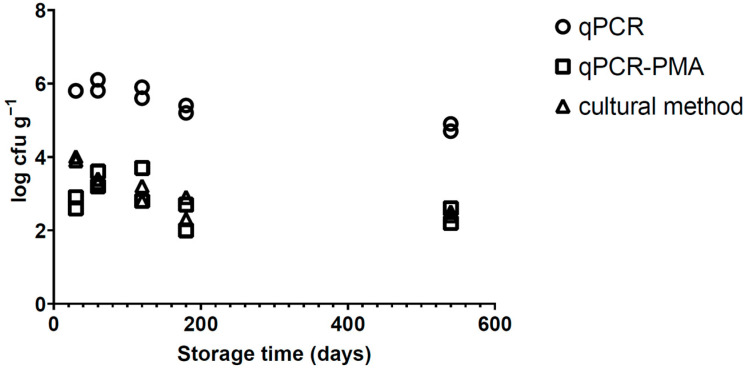
Quantification of *Salmonella* population (log cfu g^−1^) in roasted peanut kernels after storage at 28 °C over 540 d by qPCR, qPCR-PMA and culture method (XLD agar). qPCR = real-time PCR; PMA = propidium monoazide. Limit of quantification = 1 log cfu g^−1^ and 41 Ct. Values obtained from two independent trials.

The enumeration of *Salmonella* over the storage time in roasted peanuts is shown in Figure 2. The initial inoculation level was 5.1 log cfu g^−1^. The Ct value obtained using qPCR without PMA ranged from 24.7 to 27.2, corresponding to 6.0 log cfu g^−1^ after 60 d and 4.8 log cfu g^−1^ after 540 d. These values were significantly different (*p* > 0.05) from those found in the qPCR-PMA and plate count for the whole storage time. On the other hand, no significant difference (*p* > 0.05) was observed between qPCR-PMA and the culture method, except on day 30, when the culture method recovered 4.0 log cfu g^−1^, while qPCR-PMA predicted 2.8 log cfu g^−1^ (*p* < 0.05). After 60 and 120 d, the qPCR-PMA and plate count showed very similar results, between 3.0 and 3.4 log cfu g^−1^ (Figure 2). In addition, a decrease in the viable cell counts was observed for both methods after 180 and 540 d, achieving a bacterial load of around 2.5 log cfu g^−1^.

**Figure 3:**
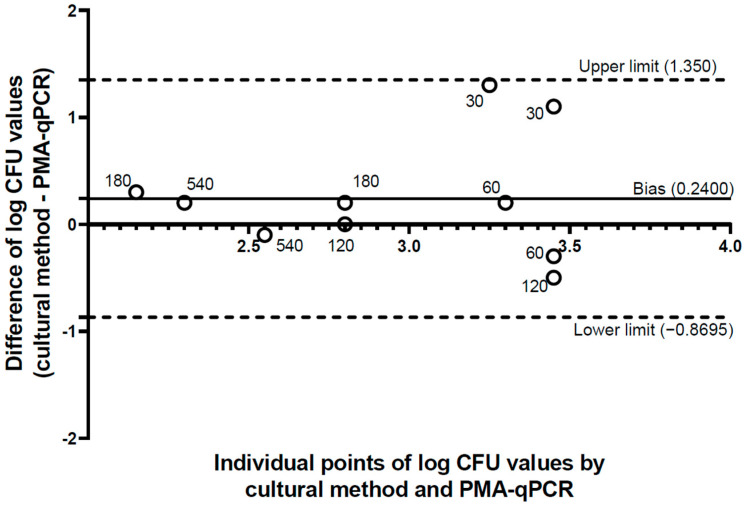
Bland–Altman analysis of the agreement between the individual values of the *Salmonella* population obtained in the culture method (log cfu g^−1^) and those predicted by qPCR-PMA from roasted peanuts stored for 30, 60, 120, 180 and 540 d. The thin solid line represents the bias, while the lower and upper 95% limits of agreement are represented as dashed lines. The bold solid line represents the zero line.

Bland–Altman analysis was used to comparatively assess the culture method and qPCR-PMA to quantify the *Salmonella* in the roasted peanut kernels throughout the storage time (Figure 3). All data remained within the 95% CI limits (limits of agreement) and very close to point 0 on the *y*-axis, with the exception of the 30 d results, indicating a high degree of agreement between the data obtained by PMA and the plate count. In addition, the mean difference (bias) calculated was a positive value (0.240), suggesting the underestimation of qPCR-PMA.

The inoculation level of the peanut-based products was 5.0, 4.9 and 6.6 log cfu g^−1^ for peanut brittle, *paçoca* and *pé-de-moça*, respectively. After 540 d of storage at 28 °C, both qPCR-PMA and the culture method did not detect the presence of *Salmonella* in *pé-de-moça*. For peanut brittle, although the pathogen count was below the quantification limit (1.0 log cfu g^−1^), it was detected in 1 g of sample by the culture method after enrichment in BPW. At the same time, a population close to 1 log cfu g^−1^ was estimated using qPCR-PMA. The greatest difference in the results was found for *paçoca*, namely 1.3 log cfu g^−1^ using qPCR-PMA versus 2.2 log cfu g^−1^ with the traditional method (*p* < 0.05). On the other hand, qPCR without PMA predicted counts > 2.8 log cfu g^−1^ for all products analyzed (Table 1).

## 4. Discussion

This is the first study that has evaluated the efficiency of the qPCR-PMA technique for the enumeration of *Salmonella* in roasted peanuts and three peanut-based products often consumed in Brazil, these being peanut kernels, *paçoca* and *pé-de-moça*. In fact, there are no data on the use of qPCR-PMA to quantify foodborne pathogens in nuts or peanuts [31,35,36].

In general, the shelf life of roasted peanuts and peanut-based candies ranges from six to 12 months [8]. The roasted peanut sampling time was chosen to simulate short-term (30 and 60 days) and long-term (120 and 180 d) storage. This is usually performed in the peanut industry. In addition, an extremely long-term storage period was analyzed in order to evaluate the sensitivity of the methods and to identify the presence of VBNC.

Both the storage time and enumeration methods showed a significant influence (*p* < 0.05) on the artificially inoculated *Salmonella* populations in the roasted peanut kernels. In addition, the results indicate that the DNA of dead *Salmonella* cells may remain on peanut samples for a long period of time. It suggests that qPCR without a DNA intercalant is not adequate for *Salmonella* quantification in LMF, since it overestimated the pathogen population (Figure 1). This has also been noted by Wolffs et al. [37] and Zhu et al. [38] in meat products.

The food safety of LMF is directly related to the ability of certain pathogens, such as *Salmonella*, to persist for long periods in these products. Usually, it leads to low levels of contamination, as only a few cells are able to remain in such an environment [39]. Nevertheless, as previously mentioned, an epidemiological survey of salmonellosis outbreaks involving LMF confirms that the infectious dose can be extremely low (0.04 NMP g^−1^) [40,41]. Thus, the use of techniques that have a low limit of quantification is essential. In our study, qPCR-PMA without an enrichment step was able to quantify a low level of contamination (1.3 log cfu g^−1^, Table 1). This is lower than that reported by Shekar et al. [26], namely 2–3 log cfu mL^−1^ for all target pathogens evaluated, and Li et al. [27] for *S.* Typhimurium in pure culture, namely 2.0 log cfu mL^−1^. Furthermore, the limit of detection of *Salmonella* can vary depending on the food matrix, from 1 to 4 log cfu mL^−1^ [42].

In addition, it is known that environmental stress conditions, such as desiccation, can cause cell injury and result in a VBNC status [43]. qPCR-PMA is claimed to detect “viable” cells, but viability can be defined in several ways, such as the presence of an intact membrane, the ability to metabolize compounds and/or the ability to maintain a proton gradient between the internal and external parts of the cell [31]. According to Nocker et al. [21], qPCR-PMA could be supplemented with compounds that show the presence of intact metabolism, since the state of the cell and cell wall permeability are not a clear indication that a cell is viable or dead. However, our results did not indicate the presence of VBNC on the roasted peanuts, even after storage for 540 d at 28 °C. In general, the count obtained by qPCR-PMA was similar to or slightly lower than that obtained with the plate count (*p* > 0.05).

Bland–Altman analysis was used as a supplementary tool to assess the level of agreement between the qPCR-PMA and culture methods. There was a high degree of agreement between the results, as most data were close to the line of equality (point 0 on the *y*-axis). Nevertheless, even though the obtained bias was small (0.240), the positive value indicated the slight underestimation of qPCR-PMA compared to the plate count, which does not suggest a physiological VBNC status for *Salmonella* inoculated into roasted peanuts at any of the storage periods analyzed. This underestimation could be related to the reduction in the viable cell signal when in a small concentration [44,45,46]; the presence of inhibitory substances in food matrices; or incorrect PCR mixtures or malfunctioning of the PCR apparatus [47]. In addition, changes in the cell membrane and turgor composition caused by desiccant stress or a damaged membrane could facilitate the penetration of PMA into *Salmonella* cells [48]. Finally, according to Wang et al. [49], the lack of objectivity in the qPCR-PMA method is evident, as the variability in the results is influenced by a series of factors, including the type of microorganism, the substrate that it is found in and the method used to inactivate or induce the VBNC state.

Peanut brittle is composed of blanched peanut kernels and caramel sauce, and *paçoca* is composed of ground peanuts, sugar and salt, while the ingredients in *pé-de-moça* are blanched peanut kernels, sugar and condensed milk. The main difference in the composition of *paçoca* compared to the other products is the presence of salt (NaCl). Therefore, it is not possible to state whether this ingredient would cause any interference in the performance of qPCR-PMA. Furthermore, peanut brittle was the only sample in which the qPCR-PMA showed 0.5 log cfu greater than the culture method, but without a significant difference (*p* > 0.05). However, as the counts were very close to the detection limit, we cannot state that they indicate the presence of VBNC.

## 5. Conclusions

Overall, qPCR-PMA displayed good performance in recovering *S.* Typhimurium from roasted peanuts. For peanut-based products, a low microbial load was observed after long-term storage. Indeed, this may have impaired the data evaluation since a significant difference (*p* < 0.05) between qPCR-PMA and the plate count was only noted for *paçoca*. Nevertheless, a low limit of quantification was determined using qPCR-PMA, suggesting the good sensitivity of this method. However, more studies are necessary to test this methodology with other *Salmonella* serotypes and LMF matrices in order to enhance its accuracy and to amplify its application across the LMF supply chain.

## Figures and Tables

**Table 1:** Quantification of *Salmonella* in peanut-based products after 540 d of storage at 28 °C using qPCR, qPCR-PMA and culture method (XLD agar).

Peanut Storage	*Salmonella* Typhimurium ATCC 14028				
	Quantification Method *				
	Ct qPCR	Predicted Log (Log cfu g^−1^)	Ct qPCR-PMA	Predicted Log (Log cfu g^−1^)	Culture Method
					(Log cfu g^−1^)
Peanut brittle	35.40 ± 3.75	2.8 ^a^ ± 1.1	39.64 ± 0.44	1.5 ^a^ ± 0.1	Presence in 1 g (<1.0)
*Paçoca*	30.20 ± 0.95	4.4 ^a^ ± 0.3	40.69 ± 0.30	1.3 ^c^ ± 0.1	2.2 ^b^ ± 0.1
*Pé-de-moça*	34.77 ± 0.36	3.0 ± 0.1	>42.00 ± 0.00	Undetectable	Absence in 1 g

Ct = threshold cycle; qPCR = real-time PCR; PMA = propidium monoazide. Limit of quantification = 1 log cfu g^−1^; limit of detection = 1 cfu g^−1^. * Values obtained from two independent trials with standard deviation. Means with different letters differ at a significance level of *p* < 0.05 in the row.

## Data Availability

The original contributions presented in this study are included in the article. Further inquiries can be directed to the corresponding author.
